# The Social and Communicational Profile of Children Identified with Autism in Ethiopia

**DOI:** 10.3390/children12121685

**Published:** 2025-12-11

**Authors:** Waganesh A. Zeleke, Elleni Damtew Asfaw, Angela Lee, Alanna King, Suzzane Long

**Affiliations:** 1 Department of Rehabilitation and Mental Health Counseling, College of Health Professionals, Virginia Commonwealth University, Richmond, VA 23284, USA; 2 Nia Foundation, Addis Ababa 1000, Ethiopia

**Keywords:** Ethiopia, autism spectrum disorder, social communication, children, collectivist culture, context to intervention

## Abstract

**Highlights:**

• Ethiopian children with autism spectrum disorder (ASD) experience heightened social and communication challenges.
• A robust social profile of Ethiopian children with ASD could expand the intervention efforts.
• Caregivers can use this data to reduce the stigma surrounding autism within their communities.

**Abstract:**

**Background:** In Ethiopia, children identified with autism spectrum disorder (ASD) and their families face substantial challenges in obtaining timely diagnosis and appropriate services, including limited public awareness, cultural stigma, and shortages of trained professionals. Understanding how social and communication difficulties manifest in children identified with ASD within Ethiopian service settings is essential for designing culturally and contextually responsive interventions. **Objectives:** This study aimed to describe the social-communication characteristics of children enrolled in two major autism-focused centers and to examine contextual influences shaping their communication profiles. **Methods:** A mixed-methods approach was employed. Quantitative data were collected from parents or guardians of 110 children using the Social Communication Questionnaire (SCQ). Qualitative data were gathered through five focus group discussions with 56 service providers. Descriptive and inferential statistical analyses were applied to SCQ scores, while thematic analysis was used to analyze qualitative transcripts. **Results:** Overall, 90% of participating children scored above the SCQ clinical cutoff of 15, indicating pronounced social and communication challenges, though these scores represent symptom patterns rather than confirmed diagnoses. No significant differences in SCQ scores were observed by age or gender. Thematic analysis identified four major contextual influences on children’s social-communicative behaviors: cultural beliefs about disability, multilingual communication environments, systemic barriers to service access, and persistent community stigma. **Conclusions:** These findings underscore the need for culturally adapted screening tools and community-based interventions that account for sociocultural and structural influences on communication. Strengthening early identification, reducing stigma, and improving service accessibility may enhance support for children with ASD in low-resource, collectivist settings.

## 1. Introduction

In Ethiopia, children identified with autism spectrum disorder (ASD) and their families face substantial barriers to timely diagnosis and effective interventions. These include limited public awareness, stigma, inadequate professional capacity, and minimal access to autism-specific services [1,2,3]. Misconceptions among caregivers and educators about the social and communication characteristics of ASD often delay recognition and treatment [4].

Although parent-led autism centers such as the Joy Center for Children with Autism and the Nehemiah Autism Center in Addis Ababa have pioneered parent-driven educational and behavioral supports, the broader evidence base documenting Ethiopian children’s social and communicative profiles remains sparse [4,5]. Understanding these profiles is critical because social interaction and communication are the defining domains of ASD [6], and their heterogeneity demands individualized intervention planning [7]. Internationally, studies show that distinct social–communication profiles are linked to differential responsiveness to therapy [8,9] and that tailored social-skills programs significantly improve peer engagement [10]. However, Ethiopian data describing how these domains manifest within local cultural and linguistic contexts have only recently begun to emerge [4].

Recent Ethiopian research provides foundational insights yet highlights persistent gaps. Liyew et al. [7] used principal component analysis of the Autism Treatment Evaluation Checklist (ATEC) to delineate social-interaction impairment patterns among children aged 4 to 16 years attending autism centers in Addis Ababa—offering the first psychometric evidence of coherent social-interaction factors in an Ethiopian sample. Complementing this, Ceccarelli et al. [10] validated the Communication Profile-Adapted (CP-A), demonstrating reliable caregiver-reported measurement of communication modes (e.g., gesture, speech, sign) and functions (e.g., requesting, social interaction) among Ethiopian children with neurodevelopmental disorders, including ASD. Parent-reported studies further reveal that delayed speech, limited peer engagement, and non-verbal communication predominate in early developmental histories [4,5,11]. Intervention research also indicates that structured social-skills training can enhance interpersonal interaction in Ethiopian school settings [11], while Liyew et al. [12] identified sensory-awareness factors linked to reduced social exploration, underscoring the interplay between sensory and communicative functioning. Collectively, these studies emphasize the need for a comprehensive, large-scale analysis integrating both social and communication domains, capturing expressive, receptive, and pragmatic aspects across Ethiopia’s diverse regions. Despite these emerging insights, no study to date has comprehensively examined the dual domains of social and communication functioning in Ethiopian children with ASD using a validated tool such as the SCQ.

The present study, therefore, addresses this gap by examining social and communicative characteristics through the social communication questionnaire (SCQ) and provider reported contextual influence. Because diagnostic capacity in Ethiopia remains limited, findings describe the social and communicative characteristics of children identified with autism as understood and recognized within Ethiopian service setting, not as formal DSM-5 diagnoses.

### Purpose

This study aimed to bridge local understandings of autism by identifying and describing the specific social and communication characteristics of children with ASD in Ethiopia and examining the cultural and environmental factors influencing these behaviors, guided by the following research questions:

1. What are the social and communicational profiles of Ethiopian children with autism spectrum disorder (ASD) enrolled in two major autism centers in Addis Ababa, as assessed by the Social Communication Questionnaire (SCQ)?
2. Do these social–communicational profiles differ by the child’s age or gender?
3. What cultural and environmental factors influence the social and communicational behaviors of children with ASD in Ethiopia?
4. What implications do these findings hold for improving diagnosis, intervention, and service provision in low-resource, collectivist cultural contexts?

## 2. Materials and Methods

### 2.1. Study Site and Sampling Procedure

The study was conducted at the primary organizations, the Joy Center for Children with Autism and the Nehemiah Autism Center, the two largest autism-focused organizations located in the capital city of Addis Ababa, Ethiopia. These are the two parent-owned Autism Centers, both of which are responsible for delivering the majority of autism services during this study. The Joy Center for Children with Autism was the first of its kind in the country, established in 2002 [13]. The center seeks to fully integrate children with ASD into their community through public awareness (e.g., dispelling myths such as autism is a curse from God), providing intervention and training (e.g., vocational training to parents), care services (e.g., educational and behavioral treatment), and support (e.g., parent support groups). In 2010, the Nehemiah Autism Center opened, which increased service provision to an additional 45 children and their families.

During this data collection, both centers enrolled more than 120 children on a full-time basis and had over 400 children on their waiting lists. Most of the children were enrolled in these centers based on what is referred to in this manuscript as an “informal identification of autism”. In this context, informal identification of autism refers to clinical judgments made by center-based professionals (including teachers, therapists, and program directors) based on caregiver reports, developmental histories, behavioral observations, and functional impairments in social communication and play. These decisions were not based on standardized diagnostic instruments such as the Autism Diagnostic Observation Schedule (ADOS-2) or Autism Diagnostic interview-Revised (ADI-R), which were not available in these settings. Diagnoses were therefore considered provisional and functional rather than formally standardized.

A convenience and purposive sampling technique was used to recruit 110 parents or guardians of children with ASD and 56 service providers were selected to participate in the study.

### 2.2. Measures and Data Collection Process

First, Institutional Review Board (IRB) approval was obtained from the leading researcher’s institution. The project was funded through an internal initiative at the researcher’s former institution. The lead research traveled to Ethiopia and worked collaboratively with the directors of the Joy Center for Children with Autism and the Nehemiah Autism Center in Addis Ababa, Ethiopia, who facilitated the research setup in accordance with the study criteria. Eligibility included parents or guardians of a child with autism spectrum disorder (ASD) who were enrolled in or waitlisted for both center- and home-based programs at the two main centers. Data were collected over three months, using three measures: surveys, focus group discussions (FGDs), and the Social Communication Questionnaire (SCQ)**.** Importantly, this study did not include standardized measures of intellectual functioning or adaptive behavior (e.g., IQ testing or Vineland Adaptive Behavior Scale), as such tools were not routinely available in these centers at the time of data collection.

#### 2.2.1. Survey

The survey consisted of demographic and open-ended questions about the child participants with autism. Parents and guardians of enrolled or waitlisted children with ASD were invited to participate in the study through the program directors at both centers. The demographic section included the children’s sex, age, and year enrolled or waitlisted in the program, as well as open-ended questions about their children’s social and communicative abilities. Systemic data on socioeconomic status, household income, parental education, or number of siblings were not collected due to logical constraints. For example, in Ethiopia, income is often informal, irregular, and difficult to quantify reliably through standard survey measures, limiting the feasibility of collecting accurate SES data. The lead researcher distributed surveys during scheduled parent meetings at the centers. A trained research assistant, a doctoral student at Addis Ababa University, facilitated completion of the questionnaires and provided clarification as needed. Both the lead researcher and research assistant are fluent in Amharic, the local language spoken by parents.

#### 2.2.2. The Social Communication Questionnaire (SCQ)

The Social Communication Questionnaire (SCQ) is a brief, 40-item parent-report screening measure that focuses on ASD symptomatology likely to be observed by a primary caregiver [14,15]. Each item in the SCQ requires a dichotomous “yes”/“no” response, and each scored item receives a value of 1 point for abnormal behavior and 0 points for the absence of abnormal behavior/normal behavior. The first item, “Is she/he now able to talk using short phrases or sentences?”—is not scored, but instead determines whether six items relating to abnormal language are assigned. Only “verbal” children (i.e., children with a “yes” response to the first question) are assigned the six items relating to abnormal language and can, thus, score a total of 0–39 points; “non-verbal” children (i.e., children with a “no” response to the first question) are not assigned the six items concerning abnormal language and can score a total of 0–33 points on the overall questionnaire.

There are two different versions of the SCQ. The SCQ Current asks respondents whether behaviors have occurred in the past 3 months. By contrast, the SCQ Lifetime references a complete developmental history and asks respondents to show whether behaviors have ever been present for questions 2–19 and whether behaviors were present at age four years—or to consider behaviors in the past 12 months if the child is not yet four years—for questions 20–40. The SCQ applies to subjects of any chronological age above 4.0 years, provided that their mental maturity is at least 2.0 years [16].

The SCQ was administrated in Amharic. Items referring culturally unfamiliar examples, such as Western childhood games (e.g., The Mulberry Bush) were explained using cultural relevant functional equivalents called መሃረሜን ያያችሁ “Meharimin Yayachu”, a local Ethiopian imitation and group play game. Parents were instructed to respond based on the presence of functionally similar behaviors rather than literal exposure to specific Western games. Both Lifetime and Current versions of the SCQ were used according to the child’s age and language ability. The questionnaire was administered individually in a quiet space at the centers to ensure privacy. Research staff guided parents through the 40 dichotomous (“yes”/“no”) items, providing culturally appropriate explanations when needed. Completed SCQs were checked for completeness before submission. The SCQ was selected to systematically characterize social–communication symptom profiles in the absence of standardized diagnostic instruments.

#### 2.2.3. Focus Group Discussions (FGD)

The FGDs were designed to gather service providers’ perspectives on the cultural and environmental factors that shape their understanding of the social and communication traits of children with autism in Ethiopia. Participants were asked to discuss and reflect on two guiding questions: (a) “What cultural and environmental factors influence the social and communicational behaviors of children with ASD in Ethiopia?” and (b) “What implications do these findings hold for improving diagnosis, intervention, and service provision in low-resource, collectivist cultural contexts?”

Five focus groups were conducted with 56 service providers (teachers, therapists, program coordinators, and parents/therapeutic care workers) at the two centers. Each group discussion lasted approximately 90 min and was moderated by the lead researcher and a trained facilitator using a semi-structured guide adapted from the Short Explanatory Model Interview (SEMI) [17]. The SEMI explores the subjects’ cultural background, nature of presenting problems, belief system, and help-seeking behaviors. FGDs were conducted in Amharic, audio-recorded with participants’ consent, and later transcribed and back-translated into English for analysis by a bilingual Ethiopian scholar (English and Amharic) with a background in literature and psychology to ensure accuracy.

### 2.3. Data Analysis

#### 2.3.1. Quantitative Analysis (Descriptive and Inferential Statistics)

Quantitative data from the social communication questionnaires were entered and analyzed using the Statistical Package for the Social Sciences (SPSS, Version 28). Descriptive analysis was employed to calculate the SCQ score, including means, standard deviations, and frequency distributions. The SCQ scores were examined against the established clinical cutoff of 15 to determine the proportion of “autism-positive” responses. To explore group differences, one-way analysis of variance (ANOVA) was conducted to compare mean SCQ scores across four age groups (4–6 years, 7–9 years, 10–12 years, >12 years). An independent-samples *t*-test was used to compare score differences between female and male students. Pearson’s correlation analyses were conducted to examine associations between age, gender, and overall SCQ scores. Significance levels were set at *p* < 0.05.

#### 2.3.2. Qualitative Analysis

Once the transcribed and translated data were completed, two individuals, a graduate student and the lead researcher, thoroughly read and reread each transcript. The team then held meetings to discuss and establish a shared understanding of participants’ responses, developing debriefing codes and trigger statements that succinctly summarized or clarified key issues. Thematic analysis followed Braun and Clarke’s [18] six-phase framework were used. These phases include: (1) familiarization with the data, (2) generation of initial codes, (3) searching for themes, (4) reviewing themes, (5) defining and naming themes, and (6) producing the report, followed by the analysis process. Two independent teams analyzed the transcripts to ensure inter-coder reliability. Discrepancies were discussed and resolved through consensus. Codes were organized into broader categories that reflected cultural, environmental, familial, and educational factors shaping the social and communicational behaviors of children with ASD. Final themes were refined to capture both convergent and divergent perspectives across parent and service-provider groups. Representative quotes were selected to illustrate key themes and enhance the trustworthiness of the findings.

### 2.4. Ethical Statement

Ethical approval for this study was obtained from the U.S. University’s Institutional Review Board (IRB) committee. The nature and scope of the research were explained to each participant before the study commenced. Participants were informed that their contribution was voluntary and that they would not face retribution for withdrawing from the study at any time. All participants had the opportunity to ask questions before the survey and questionnaires were compiled, or before the FGD began. They were also advised that any information would remain confidential.

## 3. Results

### 3.1. Demographic and Clinical Characteristics of the Participants

A total of 110 questionnaires were completed by parents of 85 male and 25 female students with ASD who are enrolled in both Autism Centers (see Table 1). The children ranged in age from 4 to 18 years, with a mean age of 9 (SD = 2.35). There were 20 (16 = M, 4 = F) children aged 4–6 years, 35 (30 = M, 5 = F) children aged 7–9, 30 (22 = M, 8 = F) children aged 10–12 years, and 25 (17 = M, 8 = F) children aged above 12 years. The length of treatment at the center ranged from 1 to 7 years, with a mean treatment duration of 2 years (SD = 1.95). While 88 (80%) of the children are fully enrolled in one of the centers, 21 (20%) are on the waiting list for full intervention, and their parents receive consultation services from the autism centers. Additionally, 42 service providers participated in 5 focus group discussions.

**Table 1:** Demographic and Clinical Characteristic breakdown of participants and service providers by percentage.

Participants	Characteristics	N	%
Students with Autism (N = 110)	Male	85	78
	Female	25	22
	Average age	9	(SD = 2.35)
	Average length of treatment in the center in years	2	(SD = 1.95)
Service Providers (N = 56)	Teachers/educators	32	52
	Counselors/psychologists/therapists	4	8
	Program coordinators	8	16
	Parents and Therapeutic care workers	12	24
Educational Background of Service Providers (N = 56)	M.A. and above	6	14
	Bachelor’s degree	10	24
	High school diploma	26	62

### 3.2. Quantitative Findings

#### 3.2.1. SCQ Scores and “Autism-Positive” Response

The SCQ yields a Total Score that is interpreted based on cutoff scores. Scores above the cutoff of 15 suggest the individual is likely to have ASD, and a more extensive evaluation should be undertaken [19,20,21]. We used a higher cutoff to increase specificity, as we intend to understand the children’s current daily experiences and evaluate their treatment and educational plans. To determine the “autism-positive” responses among the children enrolled in both centers, we used the clinical cutoff of 15. Table 2 shows that 90.01% of the children enrolled in the center scored 15 or higher, indicating an “autism-positive” response in almost all of them. Only 10 (9.09%) of 110 children scored below the cutoff score.

**Figure 1:**
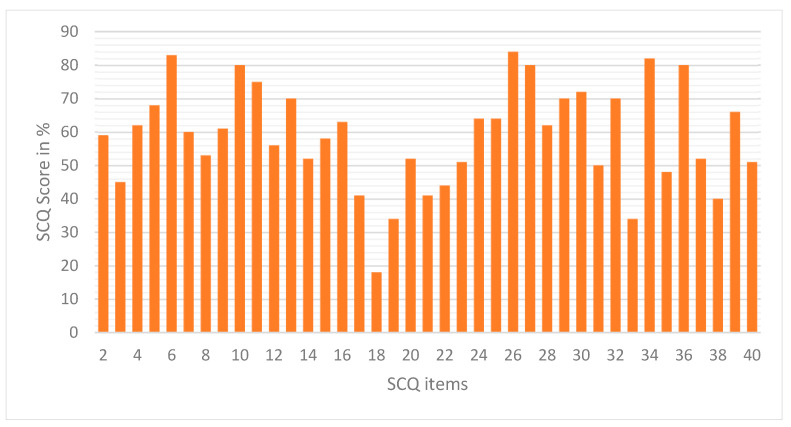
“Autism-Positive Symptom” responses in percentage. *Note: Due to space considerations, items are not spelled out here but are indicated only by their corresponding numbers from the Social Communication Questionnaire; the first author can obtain the complete list.*

We used the SCQ tool to assess the “autism-positive” symptom responses of the children because both centers employed only informal diagnostic assessment processes and did not incorporate an established, science-based protocol into their practices. Accordingly, almost all children (90.01%) had SCQ scores above the clinical cut-off, which is the score recommended for distinguishing pervasive developmental disorders from other psychiatric disorders in children (see Figure 1).

#### 3.2.2. Domain-Level Patterns

The SCQ questions 2–40 were divided into those that corresponded to three domains:

*Language and Communication domain*: This domain focuses on the child’s ability to use verbal and non-verbal communication effectively, including the ability to initiate and sustain conversations, use gestures, and exhibit delayed language development. (Questions 2, 3, 4, 5, 6, 20, 21, 22, 23, 24, 25, 34, and 35).

*Social Reciprocal Social Interaction domain*: This domain focuses on the child’s interaction with others, including the child’s enjoyment of interacting with peers, responses to social cues, and exhibited social reciprocity. (Questions 9, 10, 19, 26, 27, 28, 29, 30, 31, 32, 33, 36, 37, 39, and 40).

*Restricted, Repetitive, and Stereotyped Pattern of Behavior domain*: This domain includes questions about a child’s repetitive movements, rituals, or fixations and the impact these behaviors may have on the child’s ability to function in different environments. (Questions 7, 8, 11, 12, 13, 14, 15, and 16).

**Figure 2:**
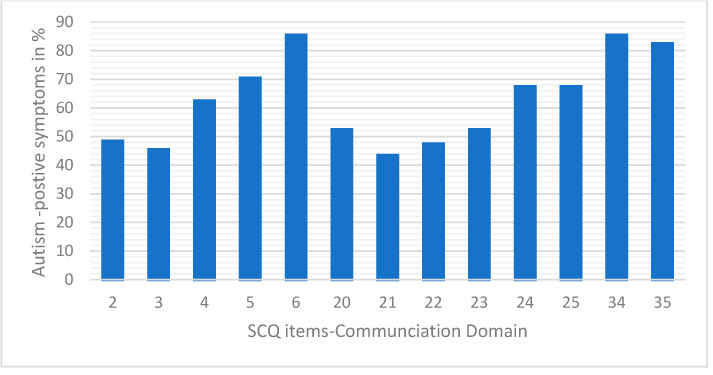
Percentage of ‘autism-positive’ responses to SCQ Communication domain items among participants.

**Figure 3:**
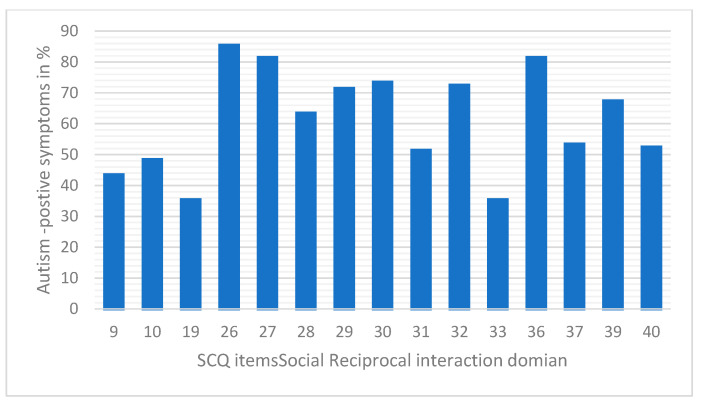
Percentage of ‘autism-positive’ responses to SCQ Social Reciprocal Interaction domain items among participants.

**Figure 4:**
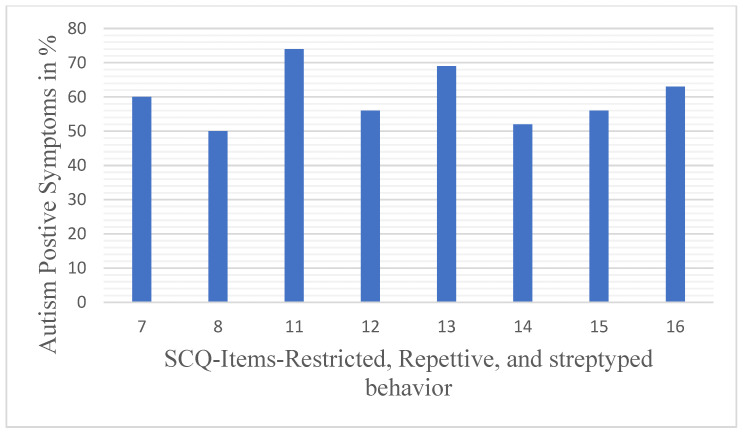
Percentage of ‘autism-positive’ responses to SCQ Restricted, Repetitive, and Stereotyped Behaviors domain items among participants.

We analyzed the three domain scores—Social Relating (Figure 2), Communication (Figure 3), and Range of Restrictive Behaviors (Figure 4)—to determine the “autism-positive symptom” response score. Variation in the response patterns observed in the Communication domain ranged from 47% to 81% of the autism-positive response pattern. The response pattern to the Social Reciprocal Interaction domain ranged from 35% to 85%, and 52% to 75% for the Restricted, Repetitive, and Stereotyped Behavior domains, respectively.

#### 3.2.3. Age and Gender Comparison

**Figure 5:**
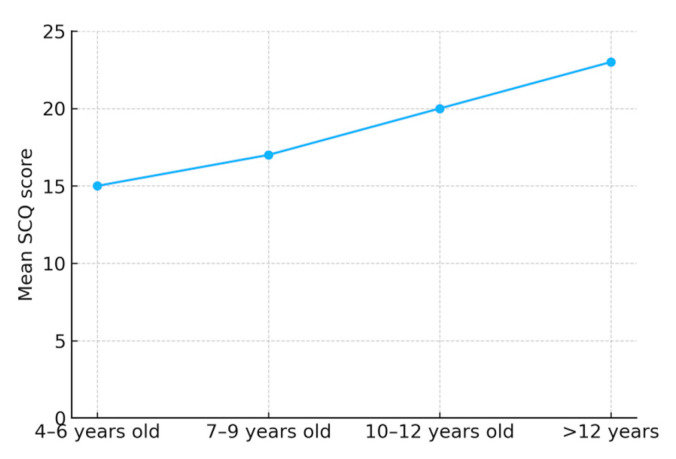
Mean SCQ score of participants based on age range.

The one-way analysis of variance revealed no significant effects of age group on SCQ scores. A Pearson’s correlation result indicates that there is no significant correlation between age and overall SCQ score (r = 0.007, *p* > 0.05) and between gender and overall SCQ score (r = 0.009, *p* > 0.05). Figure 5 shows the mean scores for the various age groups.

The percentage of the “autism-positive” responses to each of the 39 SCQ items was examined in all of the completed questionnaires. Considerable variability in the individual questions of the SCQ items was observed. For example, this varied from 15% score for the item: “Has she/he ever had any objects (other than a soft toy or comfort blanket) that she/he had to carry around?” to 85% for items such as: “When she/he was 4 to 5, did she/he usually look at you directly in the face when doing things with you or talking to you?”; “Has she/he ever used words that she/he seemed to have invented or made up her/himself; put things in odd indirect ways; or used metaphorical ways of saying things?”, and “When she/he was 4 to 5, did she/he ever spontaneously join in and try to copy the actions in social games, such as The Mulberry Bush or London Bridge Is Falling Down?” Only 1% of the participants were scored for the following items: “Has she/he ever used your hand like a tool as if it were part of her/his own body?” and “When she/he was 4 to 5, did she/he smile back if someone smiled or laughed at her/him?”

Our findings revealed no significant difference in the “autism-positive” response rate between male and female students, which is consistent with previous studies [21]. No significant effect of age on the mean SCQ scores was found in this study; however, older children had higher mean SCQ scores than younger children. Our findings are supported by those of Aldosari et al. [22], Corsello et al. [23], Liu et al. [24], and Lee et al. [25]. Their studies suggest that younger children may have lower SCQ scores than older children, and that a lower cut-off point for identification could be used for younger children.

Our results show that most “autism-positive” symptoms, assessed using the selected SCQ questions corresponding to the ADI-R Communication, Social, and Behavior domains, were found at a relatively high frequency.

The primary purpose of using SCQ in this study was to assess the children’s clinical characteristics, as most of them entered the center without a formal diagnostic process. The SCQ manual states that the questionnaire identifies rare symptoms in the unaffected population; hence, validation studies were performed in clinical populations [26]. Although the results of this study do not endorse the diagnostic impression of the participating children, they support the presence of “autism-positive” symptoms among participants. These findings could be utilized by agencies and practitioners in the future to develop robust diagnostic validation practices.

### 3.3. Qualitative Findings: Cultural and Environmental Influence

Thematic analysis of FGDs with service providers revealed four overarching themes*: (1) family and cultural beliefs, (2) communication and language contexts, (3) systemic barriers and service access, and (4) stigma and community attitude* that contextualize children’s social–communicational challenges in Ethiopia.

#### 3.3.1. Family and Cultural Beliefs

Participants consistently emphasized that children’s social and communication behaviors are profoundly shaped by their families’ cultural interpretations of disability. Many service providers observed that parents’ early reactions to autism symptoms are filtered through spiritual and traditional worldviews, affecting when and how families seek help. One teacher noted, “*Most families believe the child’s behavior is from an evil spirit or God’s punishment. They go to holy water or religious healers before they ever come here.*”

These beliefs often result in delayed diagnosis and limited early interactional support at home. As one therapist explained, “*Because they see the behavior as spiritual, they don’t talk or play with the child as much. Mostly, they only pray instead.*” At the same time, participants acknowledged the potentially protective role of extended family networks, which, in some cases, provide emotional and practical support that encourages social engagement. “*When grandparents accept the child, everyone starts to follow,*” one program coordinator said, illustrating how family acceptance can enhance a child’s social participation.

Collectively, these reflections suggest that family belief systems both constrain and facilitate children’s social and communication development, depending on whether autism is understood as a moral, spiritual, neurological, or developmental condition.

#### 3.3.2. Language and Communication Contexts

Ethiopia’s multilingual environment also emerged as a key influence on children’s communication patterns. Service providers reported that some of the children with ASD might face linguistic overload due to simultaneous exposure to Amharic, Afaan Oromo, Tigrinya, and English. “*At home they hear Amharic, Afan Oromo, or Tigrinya, and on TV and social media, they hear English and another language,*” one teacher explained. “*For children with autism, I wonder if this mixing confuses them. They struggle to connect words to meaning.*”

In addition, participants highlighted cultural communication norms that shape both parental expectations and clinical interpretation. For instance, limited eye contact or quiet behavior, often early indicators of ASD, may be viewed as culturally appropriate. “*In our culture, children are expected to be shy and respectful,*” one educator said. “*So parents don’t see the lack of eye contact as a problem.*” These linguistic and nonverbal norms obscure early symptoms and complicate standardized assessment tools developed in Western contexts. Service providers underscored the need for culturally adapted communication frameworks that reflect local pragmatics, gestures, and expressive styles rather than solely Western benchmarks.

#### 3.3.3. Systemic Barriers and Service Access

Participants described how environmental conditions and institutional limitations constrain opportunities for children with ASD to develop social and communication skills. The majority of autism services in Ethiopia are concentrated in Addis Ababa, with minimal government support or professional training. “We are trying to do everything,” one program coordinator said, “but with only two trained therapists for more than thirty children, it is impossible to give each child enough attention.”

Economic challenges were also identified as significant barriers to social participation. Families with limited income cannot afford consistent school attendance or transportation. “*Some parents stop bringing the child because the cost is too much,*” one psychologist shared. “*Then the progress we saw disappears.*”

Furthermore, overcrowded classrooms, lack of sensory-friendly spaces, and inadequate assistive materials reduce children’s opportunities to practice communication skills. Providers emphasized that these environmental constraints often determine whether social communication improves or stagnates. This theme underscores the intersection between structural inequities and developmental outcomes.

#### 3.3.4. Stigma and Community Attitudes

Cultural stigma was described as one of the most powerful environmental forces shaping children’s social worlds. Providers noted that negative community attitudes frequently lead families to hide their children from public spaces, reducing social learning opportunities. “*Some parents say they keep the child at home because neighbors will laugh or call names,*” one teacher said. “*The child grows up without playing with others or accessing the sunlight like his peers.*”

Stigma is often linked to moral judgments about parenting. “*When a child screams or moves differently, people say, ‘The mother spoiled him,’*” another participant noted. This social blame isolates families and discourages participation in communal or religious life, key avenues for social development in collectivist settings. Despite these barriers, some participants described early shifts in attitudes through awareness campaigns and parental advocacy. “*Now more people are starting to say ‘autism’ instead of hiding it,*” a senior therapist observed. “*Families talk about their children with pride. It gives others courage.*” Another teacher said, “*autism has the highest public awareness in Ethiopia than any other disability because of the center’s effort in creating awareness and advocating for autistic children.*” These evolving narratives point to a gradual, culturally grounded redefinition of autism within Ethiopian society, one that may expand social inclusion and communication opportunities for affected children.

## 4. Discussion

The present study is among the first to comprehensively describe the social and communicative profiles of Ethiopian children with ASD, integrating quantitative SCQ results with qualitative data from service providers. The findings highlight pronounced social–communication challenges among children receiving autism service in Addis Ababa, while also illustrating how cultural, linguistic, and structural factors shape manifestation and interpretation of these challenges.

The use of SCQ served as a critical function in this context. In the absence of standardized diagnostic instrument, the SCQ provided a systematic method for characterizing ASD-related behaviors. Because both centers relied primarily on informal diagnostic assessments without standardized, science-based protocols, the SCQ provided a structured means of identifying core social and communicational characteristics consistent with ASD. This approach aligns with evidence from other low- and middle-income countries (LMICs), where validated screening tools such as the SCQ are frequently employed as first-stage caregiver-report instruments in the absence of gold-standard diagnostic evaluations [27,28,29,30]. Accordingly, the finding that over 90% of participating children scored above the SCQ cutoff indicates a high prevalence of social and communication difficulties, consistent with global epidemiological data identifying these as core ASD domains [15,16]. However, these scores should be interpreted as indicators of symptom burden rather than definitive diagnosis.

Studies conducted in Bangladesh, Nigeria, Uganda, and Brazil demonstrate that the SCQ offers acceptable sensitivity and specificity for identifying children at risk of ASD in community and clinical settings where access to formal assessments like the ADOS-2 or ADI-R is limited [22,23,24,25,28,29,30]. Ethiopian and other African research further underscores the importance of culturally adapting and validating parent-report measures to reflect local languages, communication styles, and caregiving practices [31,32]. Collectively, these findings support the feasibility and utility of the SCQ as a cost-effective and contextually appropriate screener in low-resource, collectivist settings. Such applications can improve early detection and facilitate timely intervention, while future work should aim to integrate culturally validated diagnostic tools within Ethiopia’s expanding network of autism centers.

The qualitative results of this study also reflect that Ethiopian families face additional layers of difficulty due to the stigma associated with developmental disorders. Studies [1,3,4] found that many Ethiopian parents experience societal blame or isolation, which affects their well-being and limits the resources and support available to their children. Cultural and environmental factors play a significant role in shaping the social and communication behaviors of children with ASD. These factors can vary greatly depending on geographical location, societal norms, and available resources, and they often influence how children with ASD are perceived, diagnosed, and supported within their communities. Below are some key cultural and environmental influences. However, the lived context of Ethiopian families reveals unique cultural and systemic influences that shape both symptom manifestation and service access [33].

In many Western contexts, early social–communication challenges, such as reduced eye contact, limited gesture use, or atypical reciprocity, are viewed as salient diagnostic markers. In contrast, within Ethiopian collectivist culture, child shyness, deference to adults, and indirect gaze can be culturally appropriate behaviors, often delaying identification. Similar findings have been reported across other African and Asian societies [34,35], where local communication norms intersect with diagnostic interpretation. The multilingual environment in Ethiopia, with children simultaneously exposed to Amharic, Afaan Oromo, Tigrinya, and English, further complicates language acquisition and assessment. These findings suggest that standardized measures, such as the SCQ, must be culturally adapted to reflect local linguistic structures and pragmatic norms.

Qualitative themes indicated that parents’ initial responses to autism symptoms are often embedded in religious and traditional explanatory models. Families may attribute autism to spiritual causes, such as divine punishment or possession, consistent with prior Ethiopian studies [1,4]. These beliefs influence help-seeking behaviors—often leading families to pursue spiritual healing before professional evaluation. Yet, the collectivist nature of Ethiopian families also offers a potential protective buffer: when grandparents and extended relatives accept the diagnosis, stigma is reduced and the child’s inclusion improves. This underscores the importance of culturally grounded psychoeducation that partners with religious and community leaders to promote understanding rather than confrontation of spiritual frameworks.

The study’s findings highlight that stigma remains one of the most persistent barriers to inclusion, limiting children’s participation in social and educational environments. Families frequently withdraw their children from community spaces to avoid judgment, restricting opportunities for peer learning. This phenomenon mirrors those reported across Sub-Saharan Africa and South Asia, where stigma exacerbates caregiver stress and delays intervention [35]. Service providers identified systemic barriers as critical determinants of children’s social and communication development. Concentration of services in the capital, minimal governmental infrastructure, limited specialist training, and pervasive economic hardship severely restrict consistent intervention access. Expanding training for teachers, counselors, and community health workers could decentralize autism services, as recommended by global mental health frameworks such as the WHO’s Caregiver Skills Training (CST) initiative [1,36].

## 5. Implications for Practice, Policy, and Research

Parent-led centers, such as the Joy and Nehemiah Autism Centers, exemplify locally sustainable models that bridge service gaps. These institutions not only deliver behavioral and communication interventions but also serve as catalysts for awareness and advocacy. Scaling such models nationally could provide a cost-effective pathway toward inclusive service delivery in low-resource contexts. Policymakers can build on these community-based models by integrating autism screening into primary healthcare and training educators through continuing professional development programs [3].

For practitioners, the results suggest the importance of adapting social–communication interventions to reflect collectivist social norms, emphasizing group play, family routines, and relational interdependence. Incorporating caregivers as co-therapists, as demonstrated in global parent-mediated interventions, could enhance outcomes while mitigating the scarcity of trained professionals.

Future research should pursue longitudinal designs to examine developmental trajectories and intervention effects over time. Collaboration among universities, ministries, and autism centers could foster a national research network to inform culturally relevant screening tools. Additionally, adapting instruments such as the SCQ through cognitive interviewing in Amharic and other local languages would strengthen diagnostic validity and cross-cultural generalizability.

## 6. Strengths and Limitations

This study addresses a critical gap in the literature by providing one of the first systematic descriptions of the social and communicative profiles of children identified with autism spectrum disorder in Ethiopia, drawing on both quantitative caregiver reports and qualitative perspectives from service providers. The inclusion of two major autism focused centers and the use of a mixed-methods approach strengthen the ecological validity and contextual depth of the findings.

The findings should be interpreted in light of several limitations. First, children in this study were not diagnosed using standardized gold-standard diagnostic instruments such as the Autism Diagnostic Observation Schedule (ADOS-2) or the Autism Diagnostic Interview–Revised (ADI-R). Instead, enrollment was based on center-based clinical judgments, and the SCQ was used as a structured screening tool. As a result, the findings should be interpreted as symptom profiles rather than confirmed clinical diagnoses. Second, standardized measures of intellectual functioning, adaptive behavior, and autism severity were not available in the participating centers at the time of data collection. This limited the study’s ability to examine how symptom profiles varied by cognitive functioning or severity level. Third, systematic data on socioeconomic status, household income, parental education, and family structure were not collected. In Ethiopia, household income is frequently derived from informal, non-salaried, and unstable sources, which makes consistent and reliable quantification of income difficult in survey-based research. The absence of these variables limits understanding of how economic and household factors may influence social–communication development. Fourth, the study did not include a comparison group of typically developing children, preventing the establishment of locally normative SCQ response patterns and limiting interpretation of score specificity within the Ethiopian context. Fifth, the qualitative component relied exclusively on the perspectives of service providers. The voices of caregivers and autistic individuals themselves were not directly represented, which restricts the breadth of lived experiences captured. Finally, the study was conducted in two specialized centers in Addis Ababa, which may limit generalizability to rural regions and to families without access to specialized services.

Despite these limitations, the study provides foundational, contextually grounded data that can inform culturally responsive screening and intervention strategies. The findings underscore the urgency of locally validating ASD screening tools, developing accessible diagnostic pathways, and expanding decentralized service infrastructure. Future research should prioritize longitudinal designs, include community comparison samples, and incorporate caregiver and autistic voices to deepen cultural relevance.

## 7. Conclusions

This study provides contextually grounded insight into the social and communication characteristics of children identified with autism within Ethiopian service settings. Findings highlight the influences of cultural beliefs, multilingual environments, and systemic barriers. While the challenges are significant, the momentum created by parent-led centers and community advocacy offers an optimistic path forward. Building culturally responsive, family-centered, and evidence-based autism services in Ethiopia will require a multi-level approach that combines research, policy reform, and community empowerment. Through such collaborative efforts, Ethiopian children with autism can be better supported to thrive socially, communicatively, and educationally within their communities.

## Figures and Tables

**Table 2:** Percentage of ‘autism-positive’ SCQ responses among participants.

SCQ Score	Frequency	Percent	Cumulative Percent
0–14	10	9.09	9.09
15–33	100	90.01	100.0

## Data Availability

The original contributions presented in this study are included in the article. Further inquiries can be directed to the corresponding author.

## Abbreviations

The following abbreviations are used in this manuscript:

ASD	Autism Spectrum Disorder
SCQ	Social Communication Questionnaire
FDG	Focus Group Discussion
SEMI	Short Explanatory Model Interview
